# fNIRS as a Biomarker for Preoperative Assessment: Correlating Brain Activity with Clinical Evaluation for Lumbar Disc Herniation

**DOI:** 10.3390/bioengineering13050508

**Published:** 2026-04-28

**Authors:** Chengjie Huang, Changqing Li, Zhihai Su, Qiwei Guo, Quan Wang, Tao Chen, Yuhan Wang, Zhen Yuan, Hai Lu

**Affiliations:** 1 Department of Spinal Surgery, Fifth Affiliated Hospital of Sun Yat-Sen University, Zhuhai 519000, China; huangchj33@mail2.sysu.edu.cn (C.H.); lichq53@mail2.sysu.edu.cn (C.L.);; 2 Faculty of Health Sciences, Centre for Cognitive and Brain Sciences, Institute of Collaborative Innovation, University of Macau, Macau 999078, China; 13265092954@163.com (Z.S.);; 3 The Second Affiliated Hospital of Guangzhou Medical University, Guangzhou 510260, China; 4 Zhuhai UM Science and Technology Research Institute, University of Macau, Zhuhai 519099, China

**Keywords:** lumbar disc herniation, low back pain, functional near-infrared spectroscopy, pain matrices, biomarker, clinical assessment

## Abstract

Background: Lumbar disc herniation (LDH) is the most common etiological cause of low back pain (LBP). Objective and precise pain evaluation is of significant clinical value. Functional near-infrared spectroscopy (fNIRS) as a noninvasive neuroimaging modality, has been increasingly validated to reflect subjective pain perception through hemodynamic correlates. This study aimed to analyze the fNIRS changes in patients with LDH about to receive Unilateral Biportal Endoscopy and to further explore the feasibility of fNIRS as an objective biomarkers for clinical assessment of LDH. Methods: Resting-state fNIRS data were acquired from 67 preoperative LDH patients and 20 healthy controls (HC). Brain functional maps—including z-standardized fractional amplitude of low-frequency fluctuations (zfALFF) and seed-based functional connectivity (FC)—were extracted and quantified. Group-level comparisons were performed between LDH and HC groups across four predefined regions of interest; additionally, correlation analyses were conducted between fNIRS metrics and clinical assessment scores within the LDH cohort. Results: Compared with HC, LDH patients exhibited significantly altered zfALFF in the medial prefrontal cortex (mPFC): decreased amplitude at channel CH12 (t = −2.031, *p* = 0.045) and increased amplitude at CH21 (t = 2.462, *p* = 0.016). Whole-brain FC analysis further revealed widespread changes—particularly between the parietal somatosensory cortex and prefrontal regions. Among all tested FC–clinical indicator associations, 56 reached statistical significance after FDR correction (q < 0.05). VAS_ lumbar and SF-36_SF exhibited the highest number of significant connections. Conclusions: LDH patients with LBP exhibit notable alterations in prefrontal resting-state ALFF and FC between the parietal somatosensory cortex and prefrontal cortex relative to HC. Importantly, these neural alterations exhibit significant associations with both pain severity (VAS) and long-term health-related quality of life (SF-36), thereby strengthening their candidacy as neural correlates meriting prospective validation as objective, mechanism-informed biomarkers for clinical evaluation of lumbar disc herniation (LDH). Moreover, these findings highlight candidate neural targets for future longitudinal studies investigating early prognostic prediction and treatment response monitoring in LDH.

## 1. Introduction

Low back pain (LBP) stands as the primary cause of human disability and ranks among the major contributors to global productivity loss [1]. Lumbar disc herniation (LDH) is the leading etiological factor of LBP [2], defined as a clinical syndrome arising from the stimulation and/or compression of nerve roots and the cauda equina by herniated intervertebral disc tissue, based on the pathological basis of lumbar disc protrusion [3]. Clinical manifestations include LBP, lower extremity pain, numbness, weakness, and urinary and fecal dysfunction. LDH predominantly occurs at the L4/5 or L5/S1 spinal segments. Approximately 10% to 15% of patients fail to respond to conservative treatment and thus require further surgical management [4]. Unilateral Biportal Endoscopy (UBE) is a safe and effective minimally invasive surgical modality for LDH, yielding comparable outcomes to open surgery and micro-endoscopic discectomy [5]. Its core advantages lie in minimal invasiveness and rapid recovery: via two small incisions, an endoscope and operating instruments are inserted separately, enabling decompression or fusion procedures under clear visualization while minimizing damage to muscles and osseous structures [6].

The concept of pain matrices (PM) delineates a set of brain structures that are collectively activated by pain stimuli [7]. The classical PM encompasses a range of brain regions involved in nociceptive processing as well as those associated with pain perception during the experience of pain [8]. Functional brain imaging techniques have emerged as novel approaches for investigating the pathogenesis of pain and developing therapeutic strategies [9]. A growing body of evidence has shown that people with LBP demonstrate significantly greater declines in multiple cognitive domains than people who do not have LBP [10]. Altered activity in the cortex and neural circuits is one of the potential mechanisms.

Functional magnetic resonance imaging (fMRI) [11] and Electroencephalogram (EEG) [12,13,14] has been employed to study various pain conditions; however, this equipment is costly, bulky, and relatively fixed in location, with limitations in terms of combined usage. In contrast, functional near-infrared spectroscopy (fNIRS) offers advantages such as portability, resistance to motion artefacts, and no requirement for a magnetic environment. It enables real-time monitoring of brain tissue, providing favourable temporal and spatial resolution, and facilitates the assessment of cerebral oxygenation across multiple states, thus emerging as a focal area in research on the pathogenesis of low back pain. Previous studies [15] have demonstrated at the imaging level that long-term chronic pain can induce non-specific structural alterations in brain regions including the precuneus, superior parietal gyrus, superior temporal gyrus, and precentral gyrus. These alterations tend to normalize following effective pain treatment or relief [16,17].

Establishing an objective metric for nociception/pain represents one of the most formidable challenges in neuroscience and clinical medicine [18]. Current pain assessment techniques, which rely primarily on subjective self-reporting and clinical evaluations, risk significantly underestimating patients’ underlying pathology, ultimately resulting in suboptimal treatment [10].

In this study, we extracted the fNIRS signal power features from the prefrontal and parietal lobes of the brain. z-standardized Fractional Amplitude of Low-Frequency Fluctuations (zfALFF) reflects the “activity level” of a specific brain region during the resting state by quantifying the amplitude of blood oxygenation signals within a designated low-frequency band. Although affected by vascular factors or systemic physiological noise, the zfALFF can generally reflect the spontaneous neural activity of the brain. The functional connectivity (FC) matrix serves as a quantitative representation of the brain’s functional network, where each row and column corresponds to a specific brain region.

We performed a comparative analysis of the clinical characteristics of the LDH group, as well as the differences between the LDH group and healthy controls (HC), by examining zfALFF and FC. This study aimed to investigate the correlation between LDH and region-specific alterations in brain function by assessing brain oxygenation via fNIRS and performing clinical evaluations of the patients. We hypothesize that LDH patients will show altered zfALFF and FC in comparison to controls. Furthermore, we hypothesize that these neural metrics will be significantly correlated with clinical scale scores, such that greater neural alterations are associated with worse clinical outcomes.

## 2. Materials and Methods

### 2.1. Subjects

This study recruited patients with LDH who were admitted to the Department of Spinal Surgery at the Fifth Affiliated Hospital of Sun Yat-sen University between January and October 2025. All enrolled patients underwent clinical evaluation by attending physicians or those with higher academic titles in accordance with the 2023 Guidelines for the Diagnosis and Treatment of Lumbar Disc Herniation issued by the Spine Surgery Branch of the Chinese Medical Association, and were confirmed to have LDH. They also met the following inclusion criteria: (1) Voluntarily signed the informed consent form; (2) Aged over 18 years; (3) No gender restrictions; (4) Diagnosed with LDH based on clinical diagnostic guidelines; (5) Scheduled to undergo unilateral biportal endoscopic discectomy. Exclusion criteria were as follows: (1) History of spinal vertebral fracture, previous spinal fusion, or spinal deformity; (2) history of spinal fixation surgery; (3) pre-existing conditions that may interfere with brain functional imaging, such as stroke or mental disorders; (4) individuals ineligible for study participation, including pregnant women and minors. In patients with lumbar disc herniation, the prevalence of low back pain was P_1_ = 46.2%, while in individuals without LDH, the prevalence of low back pain was P_2_ = 11.9%. Setting the significance level α = 0.05 and type II error rate β = 0.10, the sample size for the lumbar degenerative disease group was calculated as N = 32 cases using PASS software (Version 21.0.0). Accounting for a 20% rate of loss to follow-up and refusal, a minimum of 40 participants should be enrolled in the study. A total of 67 eligible patients were identified (41 males and 26 females). Among these, 3 patients voluntarily withdrew from the surgical plan due to personal reasons and did not proceed to the intervention phase; however, their baseline fNIRS and clinical data were still included in the correlation analysis. Detailed patient information is presented in Table 1. Participants in this study were required to carefully read the research protocol and sign an informed consent form to join the experiment. Additionally, 20 healthy individuals with a similar gender ratio and age distribution to the patient group were recruited as healthy controls (HC). All HCs underwent the following screening assessments: (1) Pain status: No acute or chronic pain; no analgesics used in the past 3 months. (2) Neurological and psychiatric conditions: No past or current neurological diseases or mental disorders. (3) Medication use: No history of central nervous system-active medication use. The study was conducted in accordance with the guidelines of the Declaration of Helsinki. The experimental protocol was approved by the Fifth Affiliated Hospital of Sun Yat-sen University (No. K214-1 of the Ethics Committee of the Fifth Affiliated Hospital of Sun Yat-sen University [2024]), and the NCT number is ChiCTR2500095864.

### 2.2. fNIRS Recording

**Figure 1:**
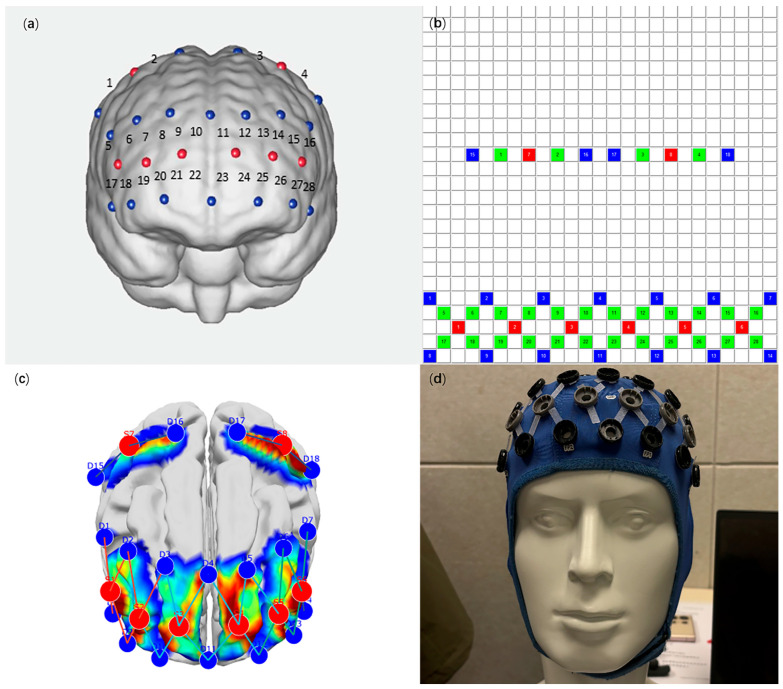
fNIRS recording illustration. (**a**) 28 detection channels 3D model; (**b**) 28 detection channels SD; (**c**) fNIRS sensitivity on brain surface; (**d**) fNIRS headset on the model. Red area indicates the sources. Blue area indicates detectors. Green area indicates detection channels.

The fNIRS data were acquired using the BrainScan near-infrared brain functional imaging system (manufactured by Beijing Psyche-Ark Science Technology Development Co., Ltd. Beijing, China), which was provided by the Brain Science Research Center of the University of Macau. This system comprises 8 light emitters and 18 detectors, forming a total of 28 detection channels; the specific channel distribution (SD) is illustrated in Figure 1. Data acquisition was performed using OBS software (OBS1.0), with laser emission wavelengths of 780/808/850 nm, a sampling rate of 36 Hz, and an acquisition duration of 5–6 min. Subsequently, the raw data were imported into MATLAB (MathWorks, Natick, MA, USA) for further analysis.

### 2.3. Experimental Procedure

Following enrollment via signed informed consent, participants were familiarized with pain assessment scales and experimental procedures under the supervision of experienced clinicians. Pain intensity and functional status were evaluated using the Visual Analogue Scale (VAS) (0 = no pain, 10 = worst imaginable pain) modified Japanese Orthopaedic Association (mJOA) score (0–30, higher scores indicating more severe pain-related impairment) and Oswestry Disability Index (ODI). Additionally, the 36-Item Short Form Health Survey (SF-36) was administered to assess health-related quality of life, encompassing eight domains: Physical Functioning (PF); Role-Physical (RP); Bodily Pain (BP), General Health (GH); Vitality (VT); Social Functioning (SF), Role-Emotional (RE), Mental Health (MH) and Reported Health Transition (HT).

**Figure 2:**
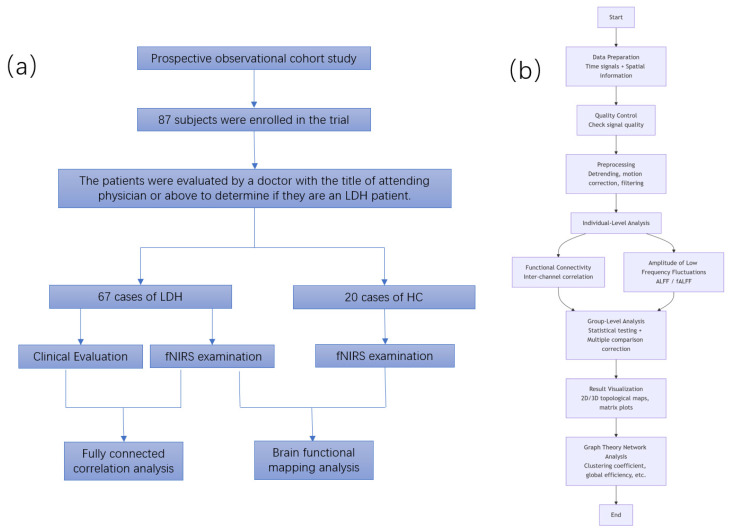
(**a**) Flowchart of experimental procedure. (**b**) fNIRS data analysis procedure.

In the present study, participants first underwent a 5 min full relaxation period in a standardized seated posture (with the back gently leaning against the chair back, both feet placed flat on the ground, hands resting naturally on the knees, and the head maintained in a neutral position) prior to fNIRS data acquisition. Concurrently, a 2 min pre-acquisition assessment was performed to maximize the reflection of their genuine neurofunctional phenotypes. Then, 5–6 min of resting-state fNIRS data was collected. Patients in the LDH group were then scheduled for UBE surgery for discectomy (nucleus pulposus removal). The HC group only underwent one fNIRS data collection of the same duration (Figure 2a).

### 2.4. fNIRS Data Analysis

fNIRS data were processed using the NIRS_KIT toolbox in MATLAB [19]. The pipeline encompassed: (1) Preprocessing: conversion of optical density to hemoglobin concentration, bandpass filtering (0.01–0.08 Hz), and motion artefact correction via Temporal Derivative Distribution Repair (TDDR) algorithm; (2) individual-level analysis: modelling the hemodynamic response using zfALFF and FC matrix to generate contrast maps for each subject; (3) group-level analysis: integrating individual contrasts for consolidated group inference (Figure 2b).

### 2.5. Statistical Analysis

Clinical evaluation and surgical data were analyzed using SPSS version 25.0 (IBM Corp, Armonk, NY, USA, 2017) to explore their intercorrelations. For brain functional maps and functional connectivity (FC) matrices, correlation analyses were conducted in MATLAB and R: specifically, comparisons of brain functional maps between the LDH and HC, as well as associations between FC matrices of the LDH group and clinical scale scores. Pearson’s correlation coefficient was employed for all correlation analyses, with false discovery rate (FDR) correction applied to account for multiple comparisons. A threshold of *p* < 0.05 was considered statistically significant. To assess the correlation between the FC matrix and the clinical scale scores, we conducted a partial correlation analysis. In the analysis, we controlled for the following variables: Pain domain: VAS_lumbar, VAS_leg, and VAS_Max (the higher value of the two VAS scores) and SF-36_BP; Neurological function domain: JOA; Functional disability domain: ODI; Quality of life domain: SF-36, including PF, RP, BP, GH, VT, SF, RE, MH and HT.

## 3. Results

### 3.1. Clinical Data and Evaluation Analysis of the Patient

Based on the standardized clinical features, the Euclidean distance matrix among participants was computed. We constructed multi-dimensional clinical distance matrices. Specifically, LSS was negatively correlated with VAS_Lumbar (t = −0.384, *p* = 0.001), while operating time showed a positive correlation (t = 0.384, *p* = 0.002); BMI was negatively correlated with VAS_Leg (t = −0.260, *p* = 0.033); ODI was negatively correlated with JOA (t = −0.730, *p* = 0.000); VAS_Lumbar was negatively correlated with VT, MH and HT respectivity (t = −0.271, *p* = 0.026; t = −0.413, *p* = 0.001; t = −0.243, *p* = 0.047); and JOA was positively correlated with a set of SF-36 indicators (*p* < 0.05). These findings demonstrate that the correlations observed between the clinical scales and clinical data collected in the present study align with prior research and clinical practice, and can effectively characterize patients’ pain, functional impairment, neurological function, and quality of life dimensions.

### 3.2. fNIRS Brain Function Differences: LDH Group and HC

**Figure 3:**
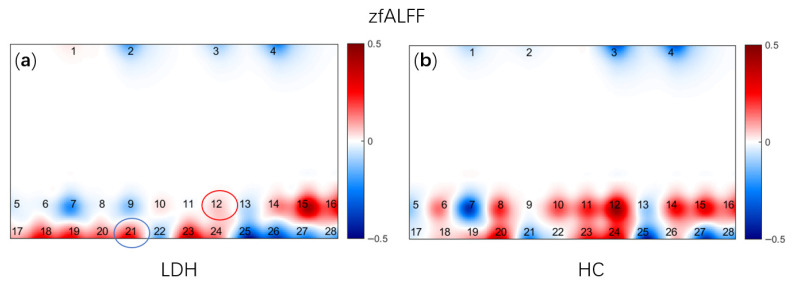
zfALFF 2D maps. (**a**) zfALFF of LDH group; (**b**) zfALFF of HC group; the red circle indicates that the HC group shows a significantly greater enhancement compared to the LDH group. The blue circle indicates that the HC group shows a significantly weaker condition compared to the LDH group.

The visualization of zfALFF derived from resting-state fNIRS brain functional maps is presented in Figure 3. Compared with the HC group, LDH group exhibited significant differences in the amplitude of spontaneous neural activity within the medial prefrontal cortex (mPFC) (CH12: t = −2.031, *p* = 0.045; CH21: t = 2.462, *p* = 0.016).

**Figure 4:**
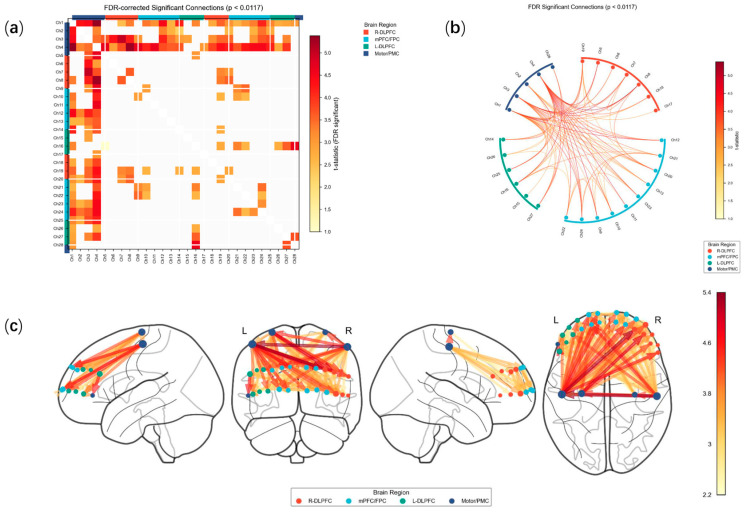
Significant FC matrix connection. (**a**) Significant independent samples *t*-test matrix. (**b**) Circular of significant connections. (**c**) Significant connections in brain region model.

An independent samples *t*-test was performed on the resting-state FCmatrix of LDH group and HC group. As illustrated in Figure 4, extensive significant differences were observed in the FC between the motor/premotor cortex (PMC) region and prefrontal cortex (PFC) region between the two groups.

### 3.3. The Relationship Between fNIRS and Clinical Scales

**Figure 5:**
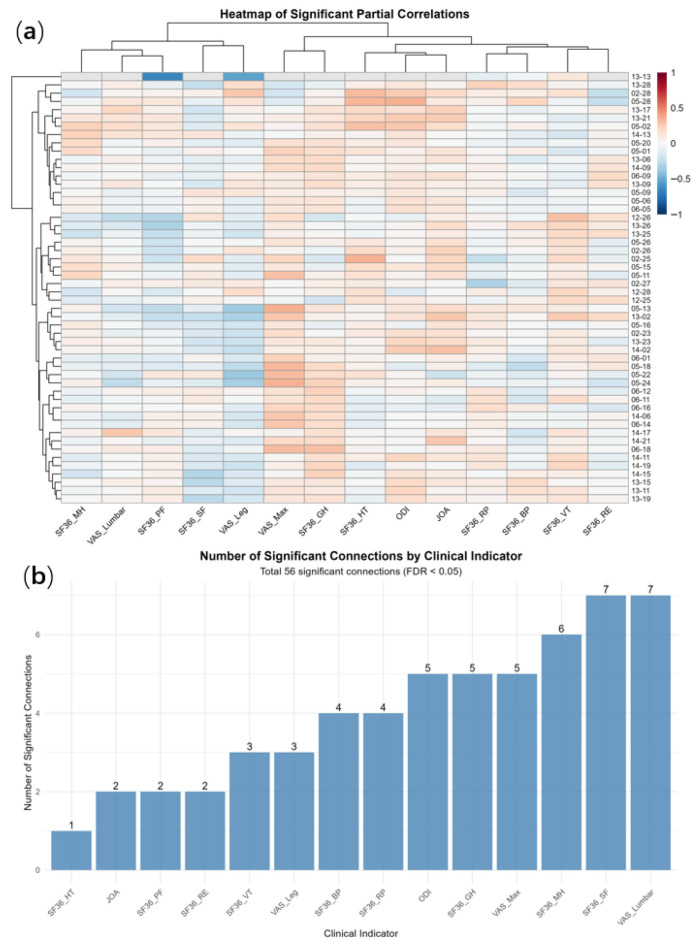
Full connection analysis of clinical indicator and FC matrix in LDH group. (**a**) Heatmap of significant partial correlations; (**b**) number of significant connections by clinical indicator. Partial correlation coefficients: These represent the net correlation between the functional connectivity and the clinical indicators after controlling for other clinical factors. Positive values indicate a positive correlation, while negative values indicate a negative correlation.

To investigate the associations between specific functional connectivity and clinical metrics while controlling for the potential confounding effects of other clinical variables, we performed a whole-connectome partial correlation analysis. Of the 10,976 partial correlation tests conducted, 8540 (77.8%) were successfully computed. FDR correction, 56 tests achieved statistical significance (FDR < 0.05), accounting for 0.66% of the total valid tests (Table A1). The top ten significant results of the analysis related to the fully connected part (FDR < 0.05) in Table 2. The overall mean of the partial correlation coefficients was 0.016 (SD = 0.140). SF-36_SF and VAS_ lumbar exhibited the highest number of significant connections, with seven each. VAS_Leg was associated with three significant connections. JOA score and SF36_HT) were linked to only two and one significant connection, respectively (Figure 5).

## 4. Discussion

In this study, we extracted the fNIRS signal power features from the PMC and PFC of the cerebral cortex. Statistical analyses were performed to examine the clinical characteristics of LDH group and the between-group differences between the LDH group and HC group. By investigating the associations between cerebral blood oxygen changes and clinical scales, our objective is to investigate the potential associations between LDL-related pain and the functional spectrum of the brain.

In prior studies, significant differences in α/β band features of EEG patterns have been observed between patients with LDH and HC, indicating potential long-term brain functional alterations in LDH patients [16]. Wang et al. [20]. investigated the cortical mechanisms underlying impaired postural control in patients with chronic low back pain (CLBP) and reported that compared with age-matched healthy individuals, CLBP patients exhibited significantly reduced postural control ability during static standing. Zeng et al. [17,21] leveraged resting-state fNIRS and supervised machine learning to identify chronic pain patients, demonstrating superior performance in detecting potential FC features. This work opens a novel avenue for the diagnosis of chronic musculoskeletal pain using fNIRS and machine learning techniques.

In studies of other disease cohorts, fNIRS also holds potential as a biomarker for the diagnosis of specific conditions [22,23,24,25,26]. Wang et al. [27] investigated the correlation between the evolution of brain functional plasticity in patients with degenerative cervical spondylosis and the severity of symptoms. The results showed that when the spinal cord was compressed, most connections within the sensory-motor network were changed, while compensatory connections were observed in the primary and secondary sensory-motor regions, subcortical regions, visual-spatial regions (including the parahippocampal gyrus), as well as within and between the brainstem and cerebellum. Qu et al. [28] integrated subjective scales with objective neurophysiological metrics (fNIRS and EEG) to systematically investigate the mechanisms underlying the effects of acupuncture therapy (AT) and motor imagery (MI) in stroke rehabilitation. Their findings revealed significant differences between the two groups in both specific EEG frequency bands and cerebral oxygenation responses across targeted brain regions. This not only validated the efficacy of AT and MI in facilitating neural function recovery but also uncovered their potential neurobiological underpinnings via multimodal data fusion. Fallgatter et al. [29] reported that prefrontal cortex neural activation was attenuated in patients with Alzheimer’s disease compared to healthy participants during both resting and task-performing states. Lee et al. [30] explored the utility of fNIRS in identifying mild cognitive impairment (MCI) and subjective memory complaints (SMC). Their results demonstrated that oxygenated HbO_2_ levels were significantly lower in patients with amnestic MCI (aMCI) than in normal controls (NC), whereas no significant difference in HbO_2_ levels was observed between patients with non-amnestic MCI (naMCI) and NC. Furthermore, HbO_2_ levels in patients with severe SMC were lower than those in NC, mild SMC, and moderate SMC groups. Ho et al. [25] employed fNIRS combined with machine learning approaches to examine treatment response in patients with major depressive disorder at the six-month follow-up. The results indicated that changes in total HbO_2_ concentration in the DLPFC before and after task performance were significantly correlated with subsequent treatment outcomes.

Our findings demonstrate that, relative to HC, patients with LDH exhibit significant alterations in resting-state ALFF within the PFC and FC between the parietal somatosensory cortex and prefrontal cortex. Notably, these neural changes are significantly correlated with patients’ pain intensity and long-term quality of life, indicating their potential as objective biomarkers for the clinical assessment of LDH. PFC plays a critical role in pain perception, modulation, and reappraisal via diverse ascending and descending neural tracts. In response to persistent chronic pain stimulation, the PFC must allocate increased neural resources to process pain-related information and potentiate pain-associated pathways. These pain-related neuroplastic alterations are potentially associated with other functions mediated by the PFC, including emotional regulation and decision-making. Specifically, without direct measures of subcortical activity, our observed network reorganizations are confined to cortical regions. Regarding whether the findings of this study have a clinically significant effect size, after careful consideration and literature review, we believe that there is currently no consensus standard in the field of neuroimaging on what constitutes a “clinically significant” effect size. Traditional neuroimaging studies were not designed to estimate effect sizes and are subject to selection bias. Recent large-sample studies have also shown that even with multimodal imaging methods, the effect sizes of intergroup differences in single-variable brain metrics between patient groups and healthy controls are actually very small. Also we cannot rule out alterations in subcortical-cortical pathways, nor can we distinguish bottom-up nociceptive transmission from top-down modulation. Consequently, the cortical network changes reported here may represent only part of the neural substrate of pain, and conclusions regarding functions involving the insula and thalamus should be drawn with caution.

Likewise, the present study has several limitations that warrant consideration:

1. Inherent constraints of fNIRS: This technique primarily captures cerebral blood flow metabolism in the cerebral cortex but lacks sensitivity to deep brain pathway connectivity. Although we used a 0.01–0.08 Hz band-pass filter, we were unable to separate low-frequency physiological noise at the same frequency as the Mayer waves, and some of the observed inter-group differences may be confounded by autonomic dysfunction. Future research may integrate additional functional imaging modalities. For example, high-density fNIRS combined with head model reconstruction, or fused with EEG, may also facilitate the indirect inference of subcortical source activities, enabling a more comprehensive investigation of disease mechanisms.

2. Complexity of cerebral cortical function: Given the coexistence of multiple neural pathways within the cortex, comorbid conditions in patients may introduce confounding biases into the study findings. This study did not collect data on the subjects’ medication use, the duration of pain, or their physical activity levels. Additionally, the anticipatory stress or anxiety that patients with lumbar disc herniation may experience while awaiting surgery could independently affect the activity of the prefrontal cortex. These factors were not included in the statistical control as potential confounding variables. Therefore, the changes in the prefrontal fNIRS signals we observed cannot be entirely attributed to pain itself. Future studies should be designed prospectively to systematically record and adjust for these variables, and incorporate psychological assessment indicators as covariates to more accurately evaluate the independent impact of the target variables.

3. Limited sample size: The clinical heterogeneity existing in the LDH group (such as concurrent lumbar spinal stenosis, multiple-level surgical history, etc.) may have confounding effects on cortical hemodynamic activity. Due to the limited sample size of this study, if further stratified by “whether there is stenosis” or “the number of surgical segments”, the sample size of each subgroup would be too small to achieve statistical robustness. Therefore, we did not conduct formal subgroup or stratified analyses to avoid the risk of false negatives or false positives. The association identified in this study between specific functional connectivity patterns and pain scores should be interpreted as hypothesis-generating rather than confirmatory in nature. In subsequent studies, we will adopt a multicenter cohort with a larger sample size.

## 5. Conclusions

In this study, we observed significant differences in resting-state ALFF within mPFC between patients with LDH and HC. Additionally, LBP intensity and SF-36_SF in the LDH group were significantly correlated with the FC matrix. These findings indicate that LDH patients with LBP exhibit notable alterations in prefrontal resting-state ALFF and FC between the parietal somatosensory cortex and prefrontal cortex relative to HC. This study identified that resting-state fNIRS functional connectivity patterns hold promise as potential neuroimaging biomarkers for reflecting pain burden and health-related quality of life in patients with LDH. More importantly, the spatial specificity of these patterns points to functional reorganization within the “prefrontal-parietal regulatory circuit”—a circuit that integrates top-down cognitive control and bottom-up sensory integration. Alterations in the connectivity strength of this circuit may represent the core neural mechanism underlying abnormal attentional resource allocation, biassed pain evaluation, and impaired subjective health perception in patients with chronic low back pain. We explicitly emphasize that the current findings are purely exploratory, and that the thresholds and models must undergo validation in independent cohorts prior to clinical application. In future research, we will establish a multi-centre clinical cohort, conduct longitudinal follow-ups of fNIRS brain functional maps and pain/health metrics before and after treatment, and employ machine learning approaches such as random forests to develop relevant predictive models.

## Figures and Tables

**Table 1:** Detailed patient information in LDH group.

	LDH		
Total case	67		
Age (years)	52.64 ± 15.13		
Height (cm)	164.12 ± 9.51		
Weight (kg)	67.19 ± 13.99		
BMI	24.80 ± 3.69		
Gender	male	41	
	female	26	
With LSS	yes	43	
	no	24	
Surgical segment	single		
	50	L2/3	2
		L3/4	3
		L4/5	30
		L5/S1	15
	multiple		
	14	L3/4-L4/5	4
		L4/5-L5/S1	10
Operating time (min)	117 ± 39.17		
Intraoperative Blood Loss (mL)	28.73 ± 18.56		

**Table 2:** The top ten significant results of the analysis related to the fully connected part (FDR < 0.05).

Number	FC Matrix	Clinical Indicator	Partial Correlation Coefficient	FDR	Sig	Related Direction
1	05-13	VAS_MAX	0.343	4.1502 × 10^−3^	**	Positive
2	13-15	SF36_SF	−0.270	<1.0 × 10^−4^	***	negative
3	13-21	ODI	0.243	<1.0 × 10^−4^	***	Positive
4	13-28	SF36_SF	−0.237	<1.0 × 10^−4^	***	negative
5	13-02	SF36_SF	−0.230	<1.0 × 10^−4^	***	negative
6	12-28	SF36_VT	0.200	<1.0 × 10^−4^	***	Positive
7	12-28	SF36_MH	−0.196	<1.0 × 10^−4^	***	negative
8	02-28	SF36_MH	−0.196	4.1502 × 10^−3^	**	negative
9	02-23	VAS_MAX	0.177	4.1502 × 10^−3^	**	Positive
10	05-20	SF36_GH	0.144	4.1502 × 10^−3^	**	Positive

Function connection number: FC_ XXX represents the XXXth function connection, corresponding to a specific connection within the 28 × 28 FC matrix. ***: FDR < 0.001 (Highly significant); **: 0.001 ≤ FDR < 0.01 (Significant).

**Figure A1:**
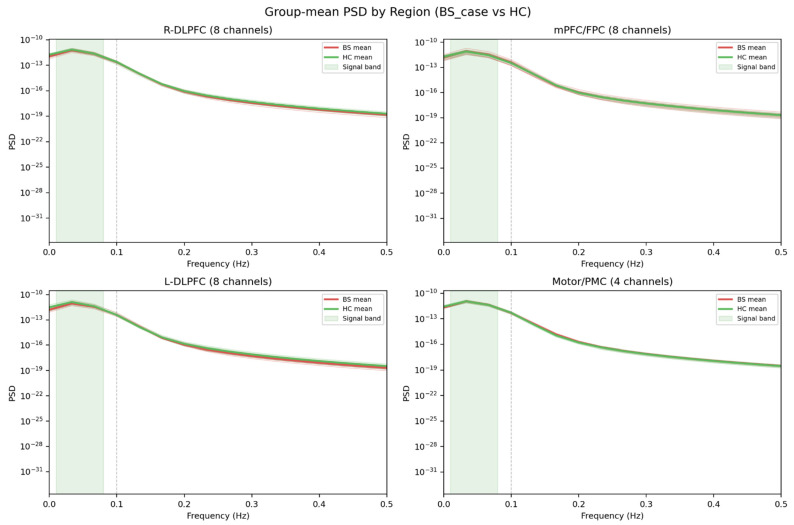
Group-mean PSD by Region.

**Figure A2:**
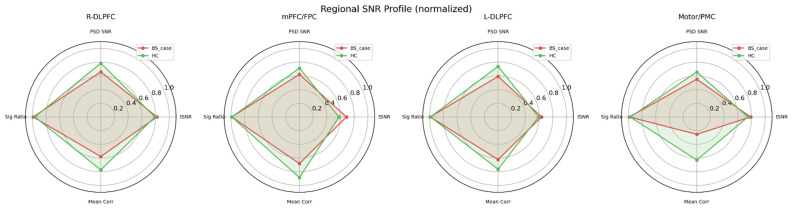
Regional SNR Profile.

## Data Availability

The original contributions presented in this study are included in this article, further inquiries can be directed to the corresponding authors.

## Abbreviations

The following abbreviations are used in this manuscript:

LBP	Low back pain
LDH	Lumbar disc herniation
PM	Pain matrices
fMIR	Functional magnetic resonance imaging
EEG	Electroencephalogram
UBE	Unilateral Biportal Endoscopy
fNIRS	functional near-infrared spectroscopy
zfALFF	z-standardized Fractional Amplitude of Low-Frequency Fluctuations
FC	Functional connectivity
PFC	prefrontal cortex
mPFC	medial prefrontal cortex
PMC	premotor cortex
CLBP	chronic low back pain
PSC	primary somatosensory cortex
DLPFC	dorsolateral prefrontal cortex
HbO_2_	oxyhemoglobin

## Appendix A

**Table A1:** The total significant results of the analysis related to the fully connected part (FDR < 0.05).

Number	FC Matrix	Clinical Indicator	Partial Correlation Coefficient	FDR	Sig	Related Direction
1	05-13	VAS_MAX	0.343	4.1502 × 10^−3^	**	positive
2	13-15	SF36_SF	−0.270	<1.0 × 10^−4^	***	negative
3	13-21	ODI	0.243	<1.0 × 10^−4^	***	positive
4	13-28	SF36_SF	−0.237	<1.0 × 10^−4^	***	negative
5	13-02	SF36_SF	−0.230	<1.0 × 10^−4^	***	negative
6	12-28	SF36_VT	0.200	<1.0 × 10^−4^	***	positive
7	12-28	SF36_MH	−0.196	<1.0 × 10^−4^	***	negative
8	02-28	SF36_MH	−0.196	4.1502 × 10^−3^	**	negative
9	02-23	VAS_MAX	0.177	4.1502 × 10^−3^	**	positive
10	05-20	SF36_GH	0.144	4.1502 × 10^−3^	**	positive
11	06-09	JOA	0.141	4.1502 × 10^−3^	**	positive
12	12-26	SF36_MH	−0.143	<1.0 × 10^−4^	***	negative
13	14-13	VAS_Lumbar	0.137	<1.0 × 10^−4^	***	positive
14	05-26	VAS_MAX	0.133	4.1502 × 10^−3^	**	positive
15	14-21	VAS_Leg	−0.133	<1.0 × 10^−4^	***	negative
16	13-26	SF36_MH	−0.137	<1.0 × 10^−4^	***	negative
17	05-06	SF36_GH	0.123	4.1502 × 10^−3^	**	positive
18	13-17	SF36_GH	0.122	<1.0 × 10^−4^	***	positive
19	05-02	VAS_Leg	0.130	4.1502 × 10^−3^	**	positive
20	13-23	VAS_MAX	0.115	<1.0 × 10^−4^	***	positive
21	14-17	SF36_PF	0.105	<1.0 × 10^−4^	***	positive
22	05-28	VAS_Lumbar	0.102	4.1502 × 10^−3^	**	positive
23	05-16	SF36_MH	0.100	4.1502 × 10^−3^	**	positive
24	05-18	SF36_SF	−0.082	4.1502 × 10^−3^	**	negative
25	14-06	SF36_RP	0.078	<1.0 × 10^−4^	***	positive
26	14-02	SF36_GH	0.075	<1.0 × 10^−4^	***	positive
27	05-15	VAS_Lumbar	0.073	4.1502 × 10^−3^	**	positive
28	06-16	SF36_VT	−0.072	4.1502 × 10^−3^	**	negative
29	06-01	SF36_MH	−0.092	4.1502 × 10^−3^	**	negative
30	14-15	SF36_BP	0.069	<1.0 × 10^−4^	***	positive
31	13-06	ODI	0.069	<1.0 × 10^−4^	***	positive
32	13-11	SF36_PF	0.093	<1.0 × 10^−4^	***	positive
33	12-25	VAS_Lumbar	−0.076	<1.0 × 10^−4^	***	negative
34	06-05	SF36_SF	0.055	4.1502 × 10^−3^	**	positive
35	14-09	ODI	0.059	<1.0 × 10^−4^	***	positive
36	13-09	SF36_BP	−0.059	<1.0 × 10^−4^	***	negative
37	02-27	VAS_Lumbar	0.063	4.1502 × 10^−3^	**	positive
38	12-28	SF36_GH	0.050	<1.0 × 10^−4^	***	positive
39	05-22	SF36_RP	−0.049	4.1502 × 10^−3^	**	negative
40	06-18	SF36_BP	−0.045	4.1502 × 10^−3^	**	negative
41	05-09	SF36_RP	0.046	4.1502 × 10^−3^	**	positive
42	13-25	VAS_Lumbar	−0.058	<1.0 × 10^−4^	***	negative
43	05-01	SF36_SF	0.048	4.1502 × 10^−3^	**	positive
44	05-24	ODI	0.037	4.1502 × 10^−3^	**	positive
45	06-14	SF36_RE	0.035	4.1502 × 10^−3^	**	positive
46	13-13	SF36_BP	−0.040	<1.0 × 10^−4^	***	negative
47	13-19	SF36_RP	0.040	<1.0 × 10^−4^	***	positive
48	05-11	ODI	0.032	4.1502 × 10^−3^	**	positive
49	13-06	SF36_VT	−0.027	<1.0 × 10^−4^	***	negative
50	02-26	SF36_RE	−0.062	4.1502 × 10^−3^	**	negative
51	02-26	SF36_SF	−0.029	4.1502 × 10^−3^	**	negative
52	06-12	SF36_HT	−0.029	4.1502 × 10^−3^	**	negative
53	02-25	VAS_Lumbar	0.020	4.1502 × 10^−3^	**	positive
54	14-11	VAS_MAX	0.011	<1.0 × 10^−4^	***	positive
55	06-11	VAS_Leg	−0.003	4.1502 × 10^−3^	**	negative
56	14-19	JOA	−0.002	<1.0 × 10^−4^	***	negative

Function connection number: FC_ XXX represents the XXXth function connection, corresponding to a specific connection within the 28 × 28 FC matrix. ***: FDR < 0.001 (Highly significant); **: 0.001 ≤ FDR < 0.01 (Significant).

## Appendix B

We have now reported exhaustive objective quality control metrics. The channel-wise signal quality was quantified using the Temporal Signal-to-Noise Ratio (tSNR) and Coefficient of Variation (CV). Across the study, signals demonstrated stable physiological viability (median tSNR: 0.027 in LDH patients vs. 0.025 in healthy controls; signal power within 0.01–0.08 Hz accounted for ~45% of total power).

Regarding exclusion criteria, each channel was evaluated for fundamental signal integrity based on the exception_channel marker embedded during preprocessing, which flags channels exhibiting macroscopic signal loss, sustained saturation, or lack of physiological modulation. Across all 87 participants (67 LDH patients + 20 healthy controls), only six individual channels across four subjects met the exception criteria and were excluded from subsequent analyses: two channels in Case 4 (CH4, CH7), one channel in Case 45 (CH3) within the patient group, and two channels in subject hanjin (CH4, CH7) and one in hujingfa (CH3) within the healthy control group. This corresponds to an exclusion rate of only 0.25% of all channel recordings (6/2, 436). Critically, no subjects were fully omitted from the generalized linear models due to excessive systemic baseline deterioration, confirming the overall robustness of the acquired dataset.

## References

[1] GBD DAII (2018). Global, regional, and national incidence, prevalence, and years lived with disability for 354 diseases and injuries for 195 countries and territories, 1990–2017: A systematic analysis for the Global Burden of Disease Study 2017. Lancet.

[2] Petersen T., Laslett M., Juhl C. (2017). Clinical classification in low back pain: Best-evidence diagnostic rules based on systematic reviews. BMC Musculoskel Dis..

[3] Knezevic N.N., Candido K.D., Vlaeyen J., Van Zundert J., Cohen S.P. (2021). Low back pain. Lancet.

[4] Hooten W.M., Cohen S.P. (2015). Evaluation and Treatment of Low Back Pain: A Clinically Focused Review for Primary Care Specialists. Mayo Clin. Proc..

[5] Tang Y., Zhang Z., Wu Z., Jin Z., Gong Y., Zhang W., Cheng K., Zhou J., Tong P., Xu T. (2025). Impact of first ambulation time on unilateral biportal endoscopy in lumbar disc herniation: A systematic review and meta-analysis. Int. J. Surg..

[6] Reis J.O.P.G., Pinto E.M., Teixeira A., Frada R., Rodrigues D., Cunha R., Miranda A. (2025). Unilateral biportal endoscopy: Review and detailed surgical approach to extraforaminal approach. EFORT Open Rev..

[7] Garcia-Larrea L., Peyron R. (2013). Pain matrices and neuropathic pain matrices: A review. Pain.

[8] Lu Y., Klein G.T., Wang M.Y. (2013). Can pain be measured objectively?. Neurosurgery.

[9] Wager T.D., Atlas L.Y., Lindquist M.A., Roy M., Woo C., Kross E. (2013). An fMRI-based neurologic signature of physical pain. N. Engl. J. Med..

[10] Zhou Z., Hui E.S., Kranz G.S., Chang J.R., de Luca K., Pinto S.M., Chan W.W., Yau S.Y., Chau B.K., Samartzis D. (2022). Potential mechanisms underlying the accelerated cognitive decline in people with chronic low back pain: A scoping review. Ageing Res. Rev..

[11] Chen W., Wagner J., Heugel N., Sugar J., Lee Y., Conant L., Malloy M., Heffernan J., Quirk B., Zinos A. (2020). Functional Near-Infrared Spectroscopy and Its Clinical Application in the Field of Neuroscience: Advances and Future Directions. Front. Neurosci..

[12] Mussigmann T., Bardel B., Lefaucheur J. (2022). Resting-state electroencephalography (EEG) biomarkers of chronic neuropathic pain. A systematic review. Neuroimage.

[13] Han Y., Zeng X., Hua L., Quan X., Chen Y., Zhou M., Chuang Y., Li Y., Wang S., Shen X. (2024). The fusion of multi-omics profile and multimodal EEG data contributes to the personalized diagnostic strategy for neurocognitive disorders. Microbiome.

[14] May E.S., Gil Ávila C., Dinh S.T., Heitmann H., Hohn V.D., Nickel M.M., Tiemann L., Tölle T.R., Ploner M. (2021). Dynamics of brain function in patients with chronic pain assessed by microstate analysis of resting-state electroencephalography. Pain.

[15] Karunakaran K.D., Peng K., Berry D., Green S., Labadie R., Kussman B., Borsook D. (2021). NIRS measures in pain and analgesia: Fundamentals, features, and function. Neurosci. Biobehav. Rev..

[16] Li R., Shao W., Zhao S., Wang L., Yu C., Liu L., Yin K. (2025). EEG alpha/beta features as a biomarker for quantifying pain in patients with lumbar disk herniation. Front. Neurosci..

[17] Zeng X., Tang W., Gao F., Tang Z., Zhang Z., Zhang J., Du M., Chen Z., Chen X., Yuan Z. (2023). Behavioral modeling and neuroimaging of impaired risky decision making in patients with chronic musculoskeletal pain. Neurophotonics.

[18] Zhang L., Chen Y., Li Z., Geng X., Zhao X., Zhang F., Bi Y., Lu X., Hu L. (2024). Advances and challenges in neuroimaging-based pain biomarkers. Cell Rep. Med..

[19] Hou X., Zhang Z., Zhao C., Duan L., Gong Y., Li Z., Zhu C. (2021). NIRS-KIT: A MATLAB toolbox for both resting-state and task fNIRS data analysis. Neurophotonics.

[20] Wang H., Liu X., Zhang G. (2025). Cortical functional mechanism of impaired postural control in patients with chronic low back pain: A fNIRS study. Chin. J. Rehabil. Med..

[21] Zeng X., Tang W., Yang J., Lin X., Du M., Chen X., Yuan Z., Zhang Z., Chen Z. (2023). Diagnosis of Chronic Musculoskeletal Pain by Using Functional Near-Infrared Spectroscopy and Machine Learning. Bioengineering.

[22] Berchansky M., Evins A.E., Evohr B., Himmelsbach Z., Pachas G.N., Karunakaran K.D., Laufer Goldshtein B., Ozana N., Gilman J.M. (2026). Detection of Δ9-Tetrahydrocannabinol Impairment Using Resting-State Functional Near-Infrared Spectroscopy: A Randomized Clinical Trial. JAMA Netw. Open.

[23] Chen L., Du B., Li K., Li K., Hou T., Jia F., Li L. (2024). The effect of tDCS on inhibitory control and its transfer effect on sustained attention in children with autism spectrum disorder: An fNIRS study. Brain Stimul..

[24] Shao K., Liu Y., Mo Y., Yang Q., Hao Y., Chen M. (2024). fNIRS-Driven Depression Recognition Based on Cross-Modal Data Augmentation. IEEE Trans. Neural Syst. Rehabil. Eng..

[25] Ho C.S.H., Wang J., Tay G.W.N., Ho R., Lin H., Li Z., Chen N. (2025). Application of functional near-infrared spectroscopy and machine learning to predict treatment response after six months in major depressive disorder. Transl. Psychiat.

[26] Rosas-Romero R., Guevara E., Peng K., Nguyen D.K., Lesage F., Pouliot P., Lima-Saad W. (2019). Prediction of epileptic seizures with convolutional neural networks and functional near-infrared spectroscopy signals. Comput. Biol. Med..

[27] Wang C., Ellingson B.M., Oughourlian T.C., Salamon N., Holly L.T. (2022). Evolution of brain functional plasticity associated with increasing symptom severity in degenerative cervical myelopathy. eBioMedicine.

[28] Qu J., Du Y., Jing J., Wang J., Bu L., Wang Y. (2025). Short-Term Longitudinal Study on Brain Network Informatics of Stroke Patients Under Acupuncture and Motor Imagery Intervention. IEEE J. Biomed. Health.

[29] Fallgatter A.J., Roesler M., Sitzmann L., Heidrich A., Mueller T.J., Strik W.K. (1997). Loss of functional hemispheric asymmetry in Alzheimer’s dementia assessed with near-infrared spectroscopy. Brain Res. Cogn. Brain Res..

[30] Lee T., Guo L., Chan A.S. (2024). fNIRS as a biomarker for individuals with subjective memory complaints and MCI. Alzheimer’s Dement. J. Alzheimer’s Assoc..

